# Does Receiving Informal Care Lead to Better Health Outcomes? Evidence From China Longitudinal Healthy Longevity Survey

**DOI:** 10.1177/01640275211052834

**Published:** 2021-11-12

**Authors:** Yixiao Wang, Wei Yang

**Affiliations:** 1 Department of Global Health and Social Medicine, 174498 King’s College London, London, UK

**Keywords:** informal care receipt, functional limitations, depressive symptoms, older people, China

## Abstract

Population aging has become a global challenge. Drawing data from Chinese Longitudinal Healthy Longevity Survey 2008, 2011, and 2014, this study examines the effect of informal care receipt on functional limitations and depressive symptoms among older people in China using lagged fixed effects model. Our findings suggest that receiving informal care is significantly associated with a slower functional decline. We also find that this effect varies across different income groups. The protective effect of informal care is more pronounced among older people with higher income compared to those with lower income. We do not observe any significant associations between receiving informal care and depressive symptoms of older people. This study highlights a pressing need for the Chinese government to establish a comprehensive long-term care system.

## Introduction

Long-term care (LTC) is important for older people who have difficulties in performing basic activities of daily living (ADL) (Hu & Li, 2018). Informal care, defined as unpaid care in daily activities provided to older people by spouse, children, grandchildren, other relatives, neighbors or friends (Van Groenou & Glaser, 2006), is the most common form of LTC in both developed and developing countries (Wang et al., 2021). In European countries, it is estimated that between 20% and 44% of LTC is provided by informal caregivers (Jang et al., 2012). In China, LTC provision is largely reliant on informal caregivers, and the proportion of informal care as accounted for total LTC provision can be as high as 90% (Du, 2015).

A growing body of studies have examined the effects of informal care on people’s health. Some researchers found that receiving help during bathing, indoor transferring, and toileting could prevent older people from accidents, receiving help in feeding could help them have a better diet and have sufficient nutrition, receiving help in taking medicine could help them better manage their health conditions (Desai et al., 2001). Informal care also provides a sense of companionship and belonging, reducing the risk of developing depression (Ji & Sun, 2018), increasing the level of life satisfaction (Wang & Li, 2011b). In contrast, others pointed out that receiving informal care may not always be associated with better health outcomes. When some of older people have to rely on other people’s help to perform everyday tasks, they may feel that they are a burden to caregivers. This negative feeling may then impact older people’s physical and mental health (Lin & Wu, 2011; Wang & Li, 2011a; Wu & Mok, 2007).

It should be noted that informal care is a type of social support embedded in specific cultural context. People from different cultural backgrounds may value informal care differently (Hu & Li, 2018). Specifically, independence for older people is socially and culturally valued in Western countries. Relying on others reduces a sense of independence, resulting in negative consequences for health (Lin & Wu, 2011). However, in most East Asian societies where Confucianism ideology is the predominant value, older people are proud of receiving care from families and feel fortunate that they can depend on family members to provide care.

Although informal care is highly valued in many East Asian societies, studies found that recent demographic and social transitions are eroding this tradition (Cong & Silverstein, 2008). Some adult children from rural areas move to urban areas for economic necessity, the long distance between them and their older parents creates a barrier for them to provide day-to-day care. Further, more and more females are participating in the labor market, which means they may not have enough time to provide long hours of care to their older parents or relatives. When they are forced to provide more care, they may experience significant physical and mental stress, which may affect the quality of care and impair their relationship with the older parents or relatives.

Studies have also found that care outcomes may vary across older people with different socioeconomic status. Informal carers from lower socioeconomic groups often have fewer learning opportunities and limited knowledge in care provision (Pampel et al., 2011). They are less likely to encourage older people to have a healthy diet and lifestyle, which is important for better health outcomes. Moreover, adult children may expect future monetary transfer such as bequest in exchange of providing care. Older people with lower income may have less savings or assets for such an exchange; therefore, they may receive less care from adult children, compared to those with higher income (Wang et al., 2021).

Against this backdrop, this study seeks to examine the effect of informal care on health of older people in China using Chinese Longitudinal Healthy Longevity Survey (CLHLS) 2008, 2011, and 2014 waves. Specifically, we ask the following research questions (RQ)

RQ1: What effect does informal care have on older people’s health trajectories over time?

RQ2: Among those receiving informal care, do longer care hours lead to better health outcomes for older people?

RQ3: Do these relationships vary across different income groups?

### Conceptual Framework

Our analysis is based on stress-buffering model which is widely used to explain the relationship between informal care and health outcomes of older people (Cohen & Wills, 1994; Suanet et al., 2020; Thoits, 2011). Older people with physical disability or mental health problems are likely to be stressed due to their circumstances. The model suggests that informal care can act as a buffer or coping strategy for older people when they receive help from spouse, adult children, other relatives and friends (Cohen & Wills, 1994). Researchers find that those who receive informal care are more likely to have a healthier lifestyle (Suanet et al., 2020). It is suggested that under the care of the informal caregivers, older people tend to be more conscious about their lifestyle or health behavior. They often change their own behaviors to match the expectations of the informal caregivers, such as giving up smoking, participating in physical exercises, and adhere to their prescription (Thoits, 2011). Evidence also shows that those receive informal care are more likely to have a healthy and nutritious diet compared to those without any care as informal caregivers are often in charge of meal preparation (Uchino, 2004).

Informal care can have significant impacts on older people’s mental health. The stress-buffering model suggests that the perception that others can provide help with daily tasks may redefine the potential stress posed by decline in health, bolster older people’s perceived ability to cope with daily activities, sustains confidence in their ability in face of challenges, and prevent psychological distress and depression (Cohen & Pressman, 2004). The receipt of informal care also produces positive effects on mental health by companionship. Although this study focuses on informal care support relating to help with preforming basic daily activities rather than emotional support, these two types of support are usually related (Cohen & Wills, 1994). Spending more time with carers who are close to them, older people’s needs for social and family interaction can be met, which distracts them from worrying about their own health, resulting in less perceived stress. Informal care can also provide a sense of belonging. It implies acceptance by one’s family members, relatives, friends and others, whom individuals are emotionally tied to and whom they view as important or influential in their lives (Cobb, 1976).

The stress buffering model explains why informal care may help to improve older people’s health outcomes, but evidence from the literature is not consistent. Some researchers suggest that informal care receipt may be linked to negative processes, such as feelings of dependence or becoming a burden (Uchino et al., 2016). Older people may find themselves have poor competence in daily life and have to rely on others. Thus, they may have lower self-confidence and self-esteem. These negative consequences may impair the relationship between older people and their caregivers, reduce the quality of care, and ultimately contribute to poor health outcomes.

The mixed evidence from the literature highlights the importance of the sociocultural context where informal care is influenced and shaped. In Western countries, people are influenced by a more individualistic culture, where the main goal of the development is to be relatively independent. Receiving informal care may reduce the independence of older people and their autonomy. Some of them even regard informal care as unhelpful assistance and are not willing to accept it (Bai et al., 2016). In contrast, in many East Asian cultures which are strongly influenced by familism and filial piety, family members are striving to fulfill filial obligations, and older people place a higher value on the role of parents in the family than they do on their own sense of independence. Thus, we hypothesize that informal care will have positive effects on older people’s health in the context of China. This leads to the first hypothesis (H):

H1: Receiving informal care is associated with a slower decline in functional and mental health among older people in China.

The relationship between intensity of care and health outcomes is not clear from the literature. In China, due to urbanization and migration over the past few decades, many adult children have moved to urban areas for economic reasons, leaving their older parents behind. Providing long hours of care may lead to interruptions at work, loss or reduced productivity, as well as limited leisure time (Cook & Dong, 2011). These difficulties may contribute to physical and mental health issues, affect the quality of informal care, and interfere with the buffering effects of informal care (Reinhard et al., 2008). Therefore, the second hypothesis is summarized as follows:

H2: Increased hours of informal care may not always lead to better health outcomes.

We predict that the protective effect of informal care is sensitive to individual income. The role of informal care as stress buffer may vary across different income groups (Krause & Borawski-Clark, 1995). Compared with those with high-income, older people with lower income are often less likely to have a healthy lifestyle which is important for their health (Thoits, 2011). They may also have limited financial resources to offer compensation to their informal carers in exchange of care. Therefore, they may receive less care or poor quality of care from their children, compared to those with higher income. Without sufficient financial compensation, informal carers may feel stressed and burdened by older people, which will affect quality of care and consequently health outcomes of the older people. Therefore, the third hypothesis is formulated as follows:

H3: The effects of informal care on health outcomes are more pronounced among older people with higher income, compared those with lower income.

## Methods

### Data and Sample

Individual-level data are drawn from the 2008, 2011, and 2014 waves of the CLHLS, a nationally-representative interview survey of healthy longevity in China (Zeng, 2004). Following the panel design, the CLHLS began in 1998 and was conducted in randomly selected sample of approximately half of the total number of counties and cities of the 22 provinces (Zeng, 2004). It collected information on sociodemographic characteristics, physical and mental health status, chronic diseases, family and social supports, and health behaviors. Although the CLHLS started to collect information on informal care from 2005, the number of survivors in the 2005, 2008, 2011, and 2014 waves is limited. Thus, our study sample encompasses older people aged 65 and above who survived in the 2008, 2011, and 2014 waves.

In the CLHLS, question on informal care is independent of question on nursing care. Respondents were asked whether they live in nursing homes. No matter whether they live in nursing home or not, they were also asked to choose their primary caregiver from the following choices: spouse, children, grandchildren, other relatives, friends, neighbors, social services, or housekeepers. Although some nursing home residents receive informal care at the same time, we cannot differentiate the effect of informal care from the effect of nursing home care in the analysis. Therefore, we exclude individuals who lived in nursing homes (*N* = 114) from the sample to reduce the potential bias to our findings. Moreover, because formal home- and community-based care is in the initial stage of development in China, number of individuals using formal home- and community-based LTC as the primary source of care is too small to do statistical analysis (*N* = 81). We exclude these individuals to focus on the effect of informal care. The final sample size of this analysis is 4396. Table 1 shows the descriptive statistics of the study sample.

**Table 1.** Descriptive Characteristics of the Sample.

Variables	Mean (SD)/Percentages
Receiving Informal Care	
No	61.62 (*N* = 2709)
Yes	38.38 (*N* = 1687)
Hours of informal care	3.209 (1.276)
Number of ADL limitations	0.377 (1.140)
Depressive symptoms	11.352 (3.330)
Age	
65–79	44.92
80+	55.08
Gender	
Female	53.88
Male	46.20
Education attainment	
Illiteracy	80.66
Elementary school	16.56
Middle school and above	2.78
Marital status	
Others	3.02
Widowed	52.27
Married	44.72
Residence	
City	16.36
Town	30.40
Rural	53.25
Living with family members	
No	17.60
Yes	82.40
Household per capita income	10921.96 (14933.49)
Smoking	
No	79.80
Yes	20.20
Drinking	
No	81.73
Yes	18.27
Self-rated Health	
Bad	18.70
Fair	35.06
Good	46.23
Number of chronic diseases	1.151 (1.317)
Cognitive function score	24.628 (7.295)
*N*	4396

Note. The unit of this study sample is the individual. Mean (SD) is presented for continuous variables, and Percentages is presented for categorical variables. ADL = activities of daily living.

### Variable Specification

#### Dependent Variable

The outcomes of interest are functional limitations and depressive symptoms. Functional limitations are captured by number of ADL limitations. In the CLHLS, there were six indicators assessing an individual’s functional limitations, that is, eating, dressing, indoor mobility, bathing, toileting, and continence. Number of ADL limitation is measured based on the number of these activities the individual is unable to perform or experience some difficulties with.

Five items were used to indicate depressive symptoms in existing studies based on CLHLS data. Out of the five questions, two measured positive feelings, for example, “Do you look on the bright side of things?”, and the other three measured negative effects, for example, “Do you often feel anxious or fearful?” The respondents were asked to choose from five answers of “Always,” “Often,” “Sometimes,” “Seldom,” and “Never.” A score from 1 to 5 was assigned to each answer, with a higher score indicating the higher level of feeling negative. Therefore, the summed score ranged from 5 to 25, with higher value indicating being more depressed.

### Independent Variable

Two key independent variables of interest are used in this study. The first independent variable of interest is a binary variable which records a value of 1 if an individual receives informal care as the primary source of care and a value of 0 if an individual does not receive any care. Focusing on those primarily rely on informal care, the second variable, intensity of informal care, is constructed based on number of hours of informal care received in last week. In all models, we use the logarithmic form of this continuous variable to account for non-linearities (Wooldridge, 2012).

### Covariates

We include the below covariates in the analysis. First, per capita income is a continuous variable measured by the question, “What was the total income of your household last year?” Income in 2008 and 2011 are inflated to 2014 values using Consumer Price Indexes. Household size and demographic composition were taken into consideration to adjust household per capita income using the Equivalent Scale (Citro & Michael, 1995). It follows the form $AE={\left(A+PK\right)}^{F}$ , where A is the number of adults in the household, K is the number of children in the household, P is the proportion of a child treated as an adult, and F is the scale economy factor that converts these adult equivalents into comparable units in terms of their efficient use of family’s resources. In this study, P is .3, F is .75 (Citro & Michael, 1995). In all models, we use the logarithmic form of household income to account for non-linearities (Wooldridge, 2012).

Based on the stress-buffering model and existing studies, we control for a set of demographic variables, health needs variables, and non-need variables in the analysis (Hu & Li, 2018; Lin & Wu, 2011). Demographic variables are age and gender. Age is a binary variable, that groups older people into two groups: 65–79 (the reference group), 80 and above. Gender is a binary variable with the female set as the reference category. Health needs variables include self-rated health, number of chronic diseases, cognitive function, smoking, and drinking. Self-rated health is a categorical variable, comprising “poor” (the reference group), “fair” and “good” status. Number of chronic diseases is a count variable representing the number of chronic diseases the respondent suffered from. Cognitive function score is a count variable measuring the number of correct answers of a total of 30 questions. These questions comprised six dimensions: orientation, registration, naming, attention and calculation, recall, and language. The total score was 30. The validity and reliability of the Chinese Mini-Mental Status Examination has been verified in many studies (Peng & Wu, 2015). Smoking and drinking are binary variables with “no” set as the reference category. Non-health variables used in the analysis are residence, education, marital status, and living arrangement. Residence comprises three groups: city (the reference group), town, and rural areas. Education is defined as no education (the reference group), elementary school, or middle school and above. Marital status is defined based on other (separated, divorced, and never married), widowed, and married. Living arrangement is a binary variable indicating whether the individual live with family members.

### Empirical Strategies

We use fixed effects panel data regression model to examine the effect of within-individual changes in informal care on within-individual changes in health (Wooldridge, 2012). Existing studies which examine the relationship between informal care and health have recognized reverse causal effects of health on care, that is, poor health may influence the receipt of informal care (Lin & Wu, 2011). For the main fixed effects model, it is possible that health decline may lead to higher possibility of receiving more informal care. In other words, the effect of informal care on functional decline may not be fully captured by the model due to the issue of reverse causality. Following the previous studies (Hu & Li, 2018; Lin & Wu, 2011), we use a lagged fixed effect model to control the health status of the individual at wave t-1, and use the informal care variable at wave t-1 to predict the health status of the individual at wave t. The specification of our model is as below

(1) $${\text{Health}}_{it}=\alpha_{0}+\alpha_{1}{\text{IFC}}_{i,t-1}+\beta_{1}{\text{Health}}_{i,t-1}+\beta_{2}X_{i,t-1}+\delta_{i}+{\text{ε}}_{\text{it}},t=2,3$$

${\text{Health}}_{it}\;$ in equation (1) denotes number of ADL limitations or depressive symptoms for an individual i at time point t . ${\text{IFC}}_{it-1}$ denotes whether receiving informal care as the primary source of care or hours of informal care for an individual i at time point t−1 . $\;\alpha_{1}\;$ denotes the relationship between informal care and health. A positive value for $\alpha_{1}\;$ indicates that informal care increases the likelihood of more ADL limitations or more depressive symptoms. A negative value for $\alpha_{1}\;$ indicates that the protective effects of informal care on health, that is, decreasing the likelihood of more ADL limitations or more depressive symptoms. $\;X_{it-1}$ denotes all other independent variables for an individual i at time point t−1 . $\delta_{i}\;$ denotes the individual-level unobserved heterogeneity.

To examine whether income modifies the relationship between informal care and health, an interaction between informal care and household per capita income is added to the model as following

(2) $${\text{Health}}_{it}=\alpha_{0}+\alpha_{1}{\text{IFC}}_{i,t-1}+\gamma_{1}{\text{IFC}}_{i,t-1}*\ln\left({\text{Income}}_{i,t-1}\right)+\beta_{1}{\text{Health}}_{i,t-1}+\;\beta_{2}X_{i,t-1}+\delta_{i}+{\text{ε}}_{\text{it}},t=2,3$$

A value of $\gamma_{1}$ in equation (2) denotes the heterogeneous effect of informal care on health across different income.

## Results

### Descriptive Statistics

Table 2 compared changes in health outcomes, that is, functional limitations and depressive symptoms from wave t-1 to wave t among older people with and without informal care. In terms of functional limitations, column 3 and 4 show mean number of ADL limitations in wave t-1 and wave t, respectively. Column 5 shows the difference in mean number of ADL limitations from wave t-1 to wave t. Our results show that those who receive informal care develop less functional limitations over time, compared to those who do not receive any informal care. In particular, those who do not receive any informal care develop on average .172 more ADL limitations ( ρ < .01) between study waves compared to those who receive some informal care. We observe similar trends for depressive symptoms. Those who receive informal care develop on average .544 ( ρ < .01) fewer depressive symptoms between study waves, compared to those who do not receive care.

**Table 2.** Changes in Health from Wave t-1 to Wave t for Among Older People with Different Status of Care Receipt in Wave t-1.

		Wave t-1	Wave t	Difference	*t*-stat
Mean number of ADL limitations	(1) Receiving no care	.012	.330	.318	27.452***
	(2) Receiving informal care	1.724	1.869	.145	2.260**
	Difference (1)–(2)			.172	2.633***
	(3) Not receiving intensive informal care	0.256	0.628	.372	13.648***
	(4) Receiving intensive informal care	1.459	1.710	.252	3.477***
	Difference (3)–(4)			.120	1.554*
Mean scores of depressive symptoms	(1) Receiving no care	11.202	11.088	−.114	−2.292**
	(2) Receiving informal care	12.145	11.487	−.658	−3.953***
	Difference (1)–(2)			.544	3.133***
	(3) Not receiving intensive informal care	11.064	11.219	.155	1.749**
	(4) Receiving intensive informal care	11.805	11.740	−.064	−.336
	Difference (3)–(4)			.220	1.038

Note. ADL = activities of daily living. Intensive informal care is defined as more than 20 hours per week (Hu & Li, 2018).

***p < .01, ** *p* < 0.5, * *p* < .1.

### Lagged Fixed Effects Model

Table 3 reports the relationship between informal care and health among older people using fixed effects model and lagged fixed effects models. These models account for differences in terms of individual characteristics in previous study wave. In terms of functional limitations, receiving informal care is associated with a significantly slower progression of functional limitations. All other things being equal, those who receive informal care in wave t-1 have less severe ADL limitations over time ( β = −1.467, ρ < .01). We then add the interaction between informal care receipt and income into the lagged fixed effects model. We find that for those receiving informal care, an additional unit increase in income significantly translates to more decrease in number of ADL limitations between study waves ( β = −.111, ρ < .05). This means that the protective effects are more pronounced among older people with higher income, compared to those with lower income. We do not find any significant effects of informal care receipt on depressive symptoms.

**Table 3.** The Relationship Between Receiving Informal Care and Health Among Older People in China.

	Number of ADL Limitations			Depressive Symptoms		
Variables	FE Model	Lagged FE Model	Lagged FE Model with Interaction	FE Model	Lagged FE Model	Lagged FE Model with Interaction
Primarily receiving informal care	1.240	−1.467	−.539	.174	−.395	−.397
	(.024)***	(.207)***	(.387)	(.130)	(.253)	(1.103)
Primarily receiving informal care* Income (ln)			−.111			.000
			(.044)**			(.128)
Income (ln)	.001	−.015	−.006	−.019	.047	.047
	(.006)	(.014)	(.014)	(.027)	(.041)	(.042)
Number of ADL limitations		.942	.949	.203	−.125	−.125
		(.159)***	(.159)***	(.055)***	(.124)	(.125)
Depressive symptoms	.009	−.010	−.011		−.011	−.011
	(.003)***	(.006)*	(.006)*		(.027)	(.027)
*N*	4396					

Notes: All the models control for health needs and non-need variables. The whole results of these models are presented in Supplementary Table 1. ADL= activities of daily living. FE= Fixed effects. Cells represents coefficient (standard error).

***p <. 01, **p <. 05, *p <. 1.

We further focus on informal care recipients, examine the relationship between hours of informal care and health, as shown in Table 4. In terms of functional limitations, there is no significant relationship between hours of informal care received in wave t-1 and number of ADL limitations in wave t. This relationship does not vary by income either. Similar trends were observed in the relationship between hours of informal care and depressive symptoms.

**Table 4.** The Relationship Between Hours of Informal Care and Health Among Older People Receiving Informal Care in China.

	Number of ADL Limitations			Depressive Symptoms		
Variables	FE Model	Lagged FE Model	Lagged FE Model with Interaction	FE Model	Lagged FE Model	Lagged FE Model with Interaction
Hours of informal care (ln)	.292	−.157	1.985	.167	−.318	−2.542
	(.054)***	(.154)	(2.525)	(.127)	(.339)	(2.371)
Hours of informal care (ln)* Income (ln)			−.111			.243
			(.157)			(.287)
Income (ln)	−.077	−.048	.642	.076	−.625	−1.352
	(.061)	(.146)	(.712)	(.138)	(.219)***	(.918)
Number of ADL limitations		.436	.845	.237	−.556	−1.080
		(.236)*	(.131)***	(.133)*	(.416)	(.513)**
Depressive symptoms	.047	.094	.119		−.012	.079
	(.026)*	(.163)	(.172)		(.132)	(.172)
*N*	1687					

Note. All the models control for health needs and non-need variables. The whole results of these models are presented in Supplementary Table 2. ADL = activities of daily living. FE = Fixed effects. Cells represents coefficient (standard error).

***p <. 01, **p < .05, *p < .1.

### Robustness Check

We perform three sets of robustness check (see Supplemental Tables 3–6). The first robustness check includes those using formal home- and community-based care as the primary source of care into the sample. We change the first independent variable, a binary variable, to a categorical variable, comprising three sets of binary variables: no care received, informal care received as primary source, and formal home- and community-based care received as primary source. The second robustness check replaces income, a continuous variable, with a categorical variable, income quintile groups. The third robustness check replaces hours of informal care, a continuous variable, with a binary variable, “whether receiving intensive informal care or not.” Following with methods from previous studies, intensive informal care is defined as more than 20 hours per week (Hu & Li, 2018). We observe similar results for both robustness checks as with our main results.

## Discussion

This study investigates the effects of informal care on health trajectories, that is, functional limitations and depressive symptoms of older people in China. This study has several new and compelling findings. We find that receiving informal care significantly slows down the progression of functional limitations, but does not have significant effect on reducing depressive symptoms. We also find that the protective effect of informal care on slower increase in functional limitations is significantly more pronounced among those with higher income compared to those with lower income. We do not observe any significant relationship between longer care hours and better health outcomes.

Our findings are consistent with existing studies in China (Hu & Li, 2018; Yang & Tan, 2019), but different from studies in Western countries (Lin & Wu, 2011). For older people in Western countries, performing daily activities with help influences their self-esteem, brings negative consequences for health (Chen & Silverstein, 2000). However, for Chinese older people, they place more importance on families than on their self-esteem. Therefore, social values in Chinese society may provide buffering effects against negative consequences, and our empirical evidence seems to support this argument.

Focusing on informal care recipients, findings that more hours of informal care do not significantly lead to less functional limitations or depressive symptoms is consistent with some existing studies (Hu & Li, 2018; Silverstein et al., 2006). One possible explanation is that informal caregivers usually lack professional knowledge and skills. When older people have severe functional limitations, solely relying on adult children may not fully satisfy their needs for professional care to maintain health. In addition, traditional pattern of family support might be weakened during the process of modernization. With rapid socioeconomic development, more adult children in rural areas migrate to developed areas for economic reasons, and this means that they may encounter great physical and psychological pressure when providing round-the-clock care, which may lower the quality of care and subsequently care outcomes.

Findings from this study have important implications for LTC system policies in China. It is crucial for the policy makers to recognize the contributions made by informal caregivers. Monetary compensations such as cash benefits should be considered to compensate the loss of productivity due to caring hours. This approach has been implemented in many aging societies. In many European countries, governments offer cash benefits to support caregivers, and cover their social security premiums and vacation pay if they provide at least 14 hours of care per week (Rhee et al., 2015). The Chinese government should consider similar policies. They should also pay more attention to those who need to provide long hours of care as intensive care provision tend to make the care relationship more strained. Respite care should be considered as an option to relieve some care burdens for the overstretched family caregivers.

Primarily relying on informal care is not a sustainable option. In many European countries, older people receive both informal and formal care at home (van Groenou & De Boer, 2016). This not only improves care quality but also share the responsibilities of caregiving and reduce the workloads of informal caregivers. However, formal home- and community-based care is still largely underdeveloped in China, especially in undeveloped areas. Government should step in to provide more comprehensive home- and community-based care services.

We find that receiving care in daily activities does not significantly reduce depressive symptoms among older people. This implies that care in daily activities cannot easily solve mental health problems, attention should be given to other types of social support for older people, such as emotional support. In the United Kingdom, communities and voluntary sections organize activities to provide professional guidance to depressed older people, which is effective in promoting social interaction and enhancing their psychological well-being (Naylor et al., 2016). There seems to be insufficient and inadequate attention paid to this vulnerable population in China. More serious mental health problems could exacerbate the risk of further impairment of daily functioning and cognition. Therefore, it is necessary to provide high-quality and timely psychological services to those in need.

Last but not least, we urge the Chinese government to pay more attention to older people with lower income. In many European countries, such as Germany, older people in lower-income groups are often provided with affordable access to formal care services (Campbell et al., 2010). China piloted its first LTC insurance program in Qingdao in 2012 to cover professional geriatric services for those with substantial or critical care needs (Du, 2015), but it is very difficult for many low-income people to afford professional care privately. The less pronounced effects of informal care and the lack of affordability of formal care are associated with a higher risk of being further decline in health. More measures should be taken to provide accessible and affordable formal care to low-income groups in China.

Some limitations of this study should be noted. Among those with informal care receipt as the primary source of care, we cannot differentiate clearly whether the individual relies only on informal care or relies on both informal and formal care as this information is not collected by the survey. Although this may result in the overestimation of the effect of informal care in this study, it should be noted that this should not change the study findings as 95% of Chinese older adults living at home are cared for only by family members (Peng & Wu, 2020). As with other longitudinal studies, CLHLS suffered from attrition resulting from both mortality and nonresponse, which may lead to sample selection bias. This may not raise major concern because earlier studies using this dataset showed that there were no systematic differences in response and attrition rates according to key characteristics (Zeng, 2004). Moreover, intensity of informal care is a comprehensive indicator, which could not be easily measured by hours of informal care. It may also depend on what and how help provided. For example, same hours of help with mobility and bathing may not reflect the same intensity, same hours of help in same tasks with different quality may not reflect the same intensity. Thus, the findings on hours of informal care should be interpreted with caution. Further information on intensity, such as hours of care in specific tasks, is needed. Based on previous studies dealing with reverse causation (Hu & Li, 2018; Lin & Wu, 2011), we use lagged fixed effects model to reduce the impact of health on informal care receipt. However, this analysis could not completely solve the issue of reversed causality. Studies in the future using alternative methods, such as instrumental variable, will be useful to identify the causal mechanisms underlying associations observed.

## Supplemental Material

sj-pdf-1-roa-10.1177_01640275211052834 – Supplemental Material for Does Receiving Informal Care Lead to Better Health Outcomes? Evidence From China Longitudinal Healthy Longevity Survey Click here for additional data file. Supplemental Material, sj-pdf-1-roa-10.1177_01640275211052834 for Does Receiving Informal Care Lead to Better Health Outcomes? Evidence From China Longitudinal Healthy Longevity Survey by Yixiao Wang and Wei Yang in Research on Aging
