# Supplementary material for: Does Receiving Informal Care Lead to Better Health Outcomes? Evidence From China Longitudinal Healthy Longevity Survey
Source: Res Aging. 2021 Nov 12;44(7-8):510–8. doi: 10.1177/01640275211052834 (PMC9284086; doi:10.1177/01640275211052834)
Supplement: sj-pdf-1-roa-10.1177_01640275211052834 – Supplemental Material for Does Receiving Informal Care Lead to Better Health Outcomes? Evidence From China Longitudinal Healthy Longevity Survey [file sj-pdf-1-roa-10.1177_01640275211052834.pdf]

Supplementary Table 1. The relationship between receiving informal care and health among older people in China

	**Number of ADL limitations**			**Depressive symptoms**		
**Variables**	**FE model**	**Lagged FE model**	**Lagged FE model with interaction**	**FE model**	**Lagged FE model**	**Lagged FE model with interaction**
**Receiving informal care**	1.240 (0.024) ***	-1.467 (0.207) ***	-0.539 (0.387)	0.174 (0.130)	-0.395 (0.253)	-0.397 (1.103)
**Receiving informal care* Income (ln)**			-0.111 (0.044) **			0.000 (0.128)
**Income (ln)**	0.001 (0.006)	-0.015 (0.014)	-0.006 (0.014)	-0.019 (0.027)	0.047 (0.041)	0.047 (0.042)
**Age**						
65-80	Ref	Ref	Ref	Ref	Ref	Ref
80+	-0.015 (0.030)	-0.071 (0.081)	-0.069 (0.081)	0.043 (0.139)	-0.059 (0.242)	-0.059 (0.242)
**Gender**						
Female	Omitted	Ref	Ref	Omitted	Ref	Ref
Male	Omitted	-0.131 (0.043) ***	-0.132 (0.043) ***	Omitted	-1.155 (0.149) ***	-1.155 (0.149) ***
**Education attainment**						
Illiteracy	Omitted	Ref	Ref	Omitted	Ref	Ref
Elementary school	Omitted	0.012 (0.035)	0.009 (0.035)	Omitted	-0.400 (0.144) ***	-0.400 (0.144) ***

Middle school and above	Omitted	0.171 (0.073) **	0.168 (0.073) **	Omitted	-0.435 (0.306)	-0.435 (0.306)
**Marital status**						
Others	Ref	Ref	Ref	Ref	Ref	Ref
Widowed	-0.054 (0.057)	-0.105 (0.137)	-0.106 (0.137)	-0.040 (0.267)	-0.167 (0.408)	-0.167 (0.408)
Married	-0.074 (0.057)	-0.030 (0.135)	-0.024 (0.135)	-0.498 (0.264) *	0.112 (0.404)	0.112 (0.404)
**Residence**						
City	Ref	Ref	Ref	Ref	Ref	Ref
Town	-0.047 (0.041)	0.354 (0.093) ***	0.351 (0.093) ***	0.629 (0.189) ***	0.035 (0.273)	0.035 (0.273)
Rural	-0.066 (0.041)	0.391 (0.095) ***	0.389 (0.095) ***	0.702 (0.192) ***	-0.118 (0.279)	-0.118 (0.279)
**Living with family members**						
No	Ref	Ref	Ref	Ref	Ref	Ref
Yes	0.095 (0.134) ***	0.030 (0.065)	0.029 (0.065)	-0.373 (0.124) ***	0.237 (0.193)	0.238 (0.193)
**Smoking**						
No	Ref	Ref	Ref	Ref	Ref	Ref
Yes	-0.069 (0.031) **	0.060 (0.072)	0.064 (0.072)	-0.104 (0.143)	0.361 (0.215) *	0.361 (0.215) *

**Drinking**						
No	Ref	Ref	Ref	Ref	Ref	Ref
Yes	0.001 (0.026)	-0.058 (0.059)	-0.060 (0.059)	-0.239 (0.121) **	0.135 (0.176)	0.135 (0.176)
**Self-rated health**						
Bad	Ref	Ref	Ref	Ref	Ref	Ref
Fair	-0.115 (0.024) ***	0.051 (0.060)	0.051 (0.060)	-1.374(0.109) ***	0.220 (0.176)	0.221 (0.176)
Good	-0.138 (0.025) ***	0.149 (0.063) **	0.152 (0.063) **	-2.434 (0.113) ***	0.529 (0.186) ***	0.530 (0.186) ***
**Number of chronic diseases**	0.014 (0.007) **	-0.012 (0.016)	-0.010 (0.016)	0.055 (0.032) *	-0.072 (0.048)	-0.072 (0.048)
**Cognitive function scores**	-0.029 (0.002) ***	0.002 (0.005)	0.002 (0.005)	-0.055 (0.008) ***	0.025 (0.014) *	0.025 (0.014) *
**Number of ADL limitations**		0.942 (0.159) ***	0.949 (0.159) ***	0.203 (0.055) ***	-0.125 (0.124)	-0.125 (0.125)
**Depressive symptoms**	0.009 (0.003) ***	-0.010 (0.006) *	-0.011 (0.006) *		-0.011 (0.027)	-0.011 (0.027)
N			4,396			

Notes: ADL= activities of daily living. FE= Fixed effects. Cells represents coefficient (standard error). *** p<0.01, ** p<0.05, * p<0.1.

Supplementary Table 2. The relationship between informal care intensity and health among older people in China

	Number of ADL limitations			Depressive symptoms		
**Variables**	**FE model**	**Lagged FE model**	**Lagged FE model with interaction**	**FE model**	**Lagged FE model**	**Lagged FE model with interaction**
**Hours of informal care (ln)**	0.292 (0.054) ***	-0.157 (0.154)	1.985 (2.525)	0.167 (0.127)	-0.318 (0.339)	-2.542 (2.371)
**Hours of informal care (ln) * Income (ln)**			-0.111 (0.157)			0.243 (0.287)
**Income (ln)**	-0.077 (0.061)	-0.048 (0.146)	0.642 (0.712)	0.076 (0.138)	-0.625 (0.219) ***	-1.352 (0.918)
**Age**						
65-80	Ref	Ref	Ref	Ref	Ref	Ref
80+	0.272 (0.383)	-0.626 (1.196)	-0.388 (1.549)	-0.697 (0.864)	3.169 (1.474) **	3.762 (1.867) **
**Gender**						
Female	Omitted	Ref	Ref	Omitted	Ref	Ref
Male	Omitted	-0.135 (0.424)	0.161 (2.302)	Omitted	-2.601 (1.589)	-2.595 (1.499) *
**Education attainment**						
Illiteracy	Omitted	Ref	Ref	Omitted	Ref	Ref
Elementary school	Omitted	-0.008 (0.658)	-0.590 (1.886)	Omitted	3.340 (1.659) **	3.214 (1.666) *

Middle school and above	Omitted	0.594 (0.846)	0.602 (0.742)	Omitted	2.891 (2.527)	0.704 (2.348)
**Marital status**						
Others	Ref	Ref	Ref	Ref	Ref	Ref
Widowed	-0.236 (0.701)	3.033 (1.923) *	3.431 (2.072) *	0.795 (1.582)	1.990 (2.583)	0.081 (1.121)
Married	0.002 (0.672)	2.382 (1.345) *	1.85 (2.185)	-0.772 (1.517)	0.661 (1.179)	-1.344 (2.346)
**Residence**						
City	Ref	Ref	Ref	Ref	Ref	Ref
Town	-0.903 (0.390) **	-0.374 (0.728)	-0.939 (1.029)	0.313 (0.889)	2.073 (1.647)	0.965 (0.643)
Rural	-0.922 (0.386) **	-0.372 (0.639)	-0.617 (0.817)	0.395 (0.880)	0.978 (0.647)	-0.042 (0.995)
**Living with family members**						
No	Ref	Ref	Ref	Ref	Ref	Ref
Yes	0.334 (0.308)	-0.034 (0.807)	-0.299 (0.925)	0.018 (0.697)	2.158 (1.127) *	1.964 (1.209)
**Smoking**						
No	Ref	Ref	Ref	Ref	Ref	Ref
Yes	-0.293 (0.375)	-0.253 (1.062)	-0.191 (1.177)	0.532 (0.846)	1.123 (1.816)	-0.661 (1.586)
**Drinking**						
No	Ref	Ref	Ref	Ref	Ref	Ref
Yes	0.131 (0.355)	0.330 (0.714)	0.620 (0.838)	-1.226 (0.799)	-0.881 (1.413)	-0.536 (1.491)
**Self-rated health**						

Bad	Ref	Ref	Ref	Ref	Ref	Ref
Fair	-0.240 (0.203)	0.157 (0.439)	0.513 (0.664)	-0.584 (0.468)	3.043 (1.124) ***	2.475 (0.902) ***
Good	-0.400 (0.232) *	0.815 (0.460) *	1.219 (0.496) **	-2.366 (0.508) ***	3.282 (1.067) ***	3.326 (1.864) ***
**Number of chronic diseases**	0.053 (0.057)	-0.174 (0.141)	0.135 (0.708)	0.266 (0.129) **	0.200 (0.241)	0.087 (0.253)
**Cognitive function scores**	-0.028 (0.013) **	0.020 (0.025)	0.065 (0.138)	-0.043 (0.028)	-0.037 (0.065)	-0.008 (0.065)
**Number of ADL limitations**		0.436 (0.236) *	0.845 (0.131) ***	0.237 (0.133) *	-0.556 (0.416)	-1.080 (0.513) **
**Depressive symptoms**	0.047 (0.026) *	0.094 (0.163)	0.119 (0.172)		-0.012 (0.132)	0.079 (0.172)
N			1,687			

Notes: ADL= activities of daily living. FE= Fixed effects. Cells represents coefficient (standard error). *** p<0.01, ** p<0.05, * p<0.1.

Supplementary Table 3. The relationship between receiving informal care and health among older people in China

	Number of ADL limitations			Depressive symptoms		
**Variables**	**FE model**	**Lagged FE model**	**Lagged FE model with interaction**	**FE model**	**Lagged FE model**	**Lagged FE model with interaction**
**Care types**						
Receiving no care	Ref	Ref	Ref	Ref	Ref	Ref
Receiving informal care	1.236 (0.023) ***	-1.117 (0.168) ***	-0.433 (0.351)	0.155 (0.128)	-0.469 (0.245) *	-0.967 (1.042)
Receiving formal care	1.454 (0.110) ***	-1.244 (0.343) ***	0.281 (0.782)	-0.324 (0.512)	0.459 (0.867)	-0.650 (2.419)
**Care types* Income (ln)**			-0.081 (0.039) **			0.059 (0.120)
**Income (ln)**	0.001 (0.006)	-0.013 (0.013)	-0.006 (0.014)	-0.017 (0.027)	0.052 (0.041)	0.047 (0.042)
**Age**						
65-80	Ref	Ref	Ref	Ref	Ref	Ref
80+	-0.006 (0.030)	-0.068 (0.077)	-0.066 (0.077)	0.061 (0.139)	-0.061 (0.241)	-0.063 (0.241)
**Gender**						
Female	Omitted	Ref	Ref	Omitted	Ref	Ref

Male	Omitted	-0.150 (0.042) ***	-0.151 (0.042) ***	Omitted	-1.145 (0.147) ***	-1.144 (0.147) ***
**Education attainment**						
Illiteracy	Omitted	Ref	Ref	Omitted	Ref	Ref
Elementary school	Omitted	0.010 (0.036)	0.008 (0.035)	Omitted	-0.402 (0.143) ***	--0.400 (0.144) ***
Middle school and above	Omitted	0.182 (0.075) **	0.178 (0.075) **	Omitted	-0.424 (0.303)	-0.422 (0.303)
**Marital status**						
Others	Ref	Ref	Ref	Ref	Ref	Ref
Widowed	-0.051 (0.057)	-0.127 (0.129)	-0.129 (0.129)	-0.055 (0.265)	-0.187 (0.405)	-0.186 (0.405)
Married	-0.078 (0.057)	-0.010 (0.128)	-0.006 (0.128)	-0.494 (0.263) *	0.076 (0.401)	0.074 (0.402)
**Residence**						
City	Ref	Ref	Ref	Ref	Ref	Ref
Town	-0.048 (0.041)	0.298 (0.087) ***	0.295 (0.087) ***	0.571 (0.186) ***	0.071 (0.270)	0.073 (0.270)
Rural	-0.066 (0.041)	0.329 (0.089) ***	0.328 (0.089) ***	0.643 (0.190) ***	-0.055 (0.276)	-0.054 (0.276)
**Living with family members**						
No	Ref	Ref	Ref	Ref	Ref	Ref

Yes	0.101 (0.026) ***	0.016 (0.061)	0.014 (0.060)	-0.393 (0.122) ***	0.244 (0.191)	0.246 (0.190)
**Smoking**						
No	Ref	Ref	Ref	Ref	Ref	Ref
Yes	-0.071 (0.031) **	0.073 (0.068)	0.076 (0.068)	-0.104 (0.143)	0.371 (0.214) *	0.368 (0.214) *
**Drinking**						
No	Ref	Ref	Ref	Ref	Ref	Ref
Yes	-0.001 (0.026)	-0.040 (0.056)	-0.041 (0.056)	-0.219 (0.120) *	0.124 (0.174)	0.125 (0.174)
**Self-rated health**						
Bad	Ref	Ref	Ref	Ref	Ref	Ref
Fair	-0.127 (0.024) ***	0.051 (0.057)	0.051 (0.056)	-1.355(0.109) ***	0.235 (0.175)	0.234 (0.175)
Good	-0.149 (0.025) ***	0.134 (0.059) **	0.136 (0.059) **	-2.419 (0.113) ***	0.551 (0.186) ***	0.550 (0.186) ***
**Number of chronic diseases**	0.014 (0.007) **	-0.017 (0.015)	-0.016 (0.015)	0.056 (0.032) *	-0.077 (0.048)	-0.077 (0.048)
**Cognitive function scores**	-0.029 (0.002) ***	-0.000 (0.005)	-0.000 (0.005)	-0.055 (0.008) ***	0.024 (0.014) *	0.024 (0.014) *

**Number of ADL limitations**		0.685 (0.129) ***	0.688 (0.129) ***	0.206 (0.055) ***	-0.066 (0.118)	-0.068 (0.118)
**Depressive symptoms**	0.009 (0.003) ***	-0.009 (0.006) *	-0.009 (0.006) *		-0.012 (0.027)	-0.011 (0.027)
N			4,477			

Notes: ADL= activities of daily living. FE= Fixed effects. Cells represents coefficient (standard error). *** p<0.01, ** p<0.05, * p<0.1.

Supplementary Table 4. The relationship between receiving informal care and health among older people in China

Variables	Number of ADL limitations			Depressive symptoms		
	FE model	Lagged FE model	Lagged FE model with interaction	FE model	Lagged FE model	Lagged FE model with interaction
**Receiving informal care**	1.239 (0.024) ***	-1.466 (0.206) ***	-0.223 （0.284）	0.170 (0.130)	-0.399 (0.253)	0.227 (0.346)
**Receiving informal care*Income**						
Receiving informal care*Lowest quintile			Ref			Ref
Receiving informal care*Second quintile			-0.379 (0.180) **			1.274 (2.363)
Receiving informal care*Middle quintile			-0.806 (0.179) ***			0.741 (0.891)
Receiving informal care*Fourth quintile			-0.570 (0.177) ***			3.577 (4.469)
Receiving informal care*Top quintile			-0.448 (0.180) **			2.695 (2.807)
**Income**						
Lowest quintile	Ref	Ref	Ref	Ref	Ref	Ref
Second quintile	-0.005 (0.023)	-0.016 (0.052)	-0.032 (0.053)	-0.121 (0.109)	0.023 (0.156)	0.262 (0.467)

Middle quintile	0.034 (0.024)	-0.034 (0.056)	0.003 (0.057)	-0.323 (0.114) ***	0.363 (0.166) **	0.171 (0.178)
Fourth quintile	-0.010 (0.027)	-0.006 (0.063)	-0.001 (0.065)	-0.349 (0.123) ***	0.350 (0.187) *	0.203 (0.378)
Top quintile	0.000 (0.027)	-0.073 (0.063)	-0.036 (0.065)	-0.266 (0.127) **	0.223 (0.189)	0.174 (0.267)
**Age**						
65-80	Ref	Ref	Ref	Ref	Ref	Ref
80+	-0.016 (0.030)	-0.067 (0.081)		0.043 (0.139)	-0.051 (0.242)	-0.059 (0.242)
**Gender**						
Female	Omitted	Ref	Ref	Omitted	Ref	Ref
Male	Omitted	-0.132 (0.043) ***	-0.095 (0.090)	Omitted	-1.161 (0.149) ***	-1.155 (0.149) ***
**Education attainment**						
Illiteracy	Omitted	Ref	Ref	Omitted	Ref	Ref
Elementary school	Omitted	0.011 (0.035)	0.009 (0.035)	Omitted	-0.421 (0.145) ***	-0.400 (0.144) ***
Middle school and above	Omitted	0.165 (0.074) **	0.168 (0.073) **	Omitted	-0.479 (0.307)	-0.435 (0.306)
**Marital status**						
Others	Ref	Ref	Ref	Ref	Ref	Ref
Widowed	-0.052 (0.057)	-0.110 (0.137)	-0.106 (0.137)	-0.052 (0.267)	-0.148 (0.408)	-0.167 (0.408)

Married	-0.072 (0.057)	-0.036 (0.135)	-0.024 (0.135)	-0.527 (0.264) **	0.148 (0.404)	0.112 (0.404)
**Residence**						
City	Ref	Ref	Ref	Ref	Ref	Ref
Town	-0.049 (0.041)	0.354 (0.093) ***	0.351 (0.093) ***	0.624 (0.189) ***	0.024 (0.273)	0.035 (0.273)
Rural	-0.069 (0.041) *	0.391 (0.095) ***	0.389 (0.095) ***	0.691 (0.192) ***	-0.131 (0.279)	-0.118 (0.279)
**Living with family members**						
No	Ref	Ref	Ref	Ref	Ref	Ref
Yes	0.095 (0.027) ***	0.028 (0.065)	0.029 (0.065)	-0.368 (0.124) ***	0.213 (0.193)	0.238 (0.193)
**Smoking**						
No	Ref	Ref	Ref	Ref	Ref	Ref
Yes	-0.069 (0.031) **	0.062 (0.072)	0.064 (0.072)	-0.116 (0.143)	0.385 (0.215) *	0.361 (0.215) *
**Drinking**						
No	Ref	Ref	Ref	Ref	Ref	Ref
Yes	0.000 (0.026)	-0.057 (0.059)	-0.060 (0.059)	-0.234 (0.121) *	0.140 (0.176)	0.135 (0.176)
**Self-rated health**						
Bad	Ref	Ref	Ref	Ref	Ref	Ref
Fair	-0.116	0.049	0.051 (0.060)	-1.365	0.224	0.221 (0.176)

	(0.024) ***	(0.060)		(0.109) ***	(0.176)	
Good	-0.139 (0.025) ***	0.147 (0.063)	0.152 (0.063) **	-2.419 (0.113) ***	0.524 (0.186) ***	0.530 (0.186) ***
**Number of chronic diseases**	0.014 (0.007) **	-0.011 (0.016)	-0.010 (0.016)	0.053 (0.032) *	-0.069 (0.048)	-0.072 (0.048)
**Cognitive function scores**	-0.029 (0.002) ***	0.002 (0.005)	0.002 (0.005)	-0.054 (0.008) ***	0.024 (0.014) *	0.025 (0.014) *
**Number of ADL limitations**		0.940 (0.157) ***	0.949 (0.159) ***	0.205 (0.055) ***	-0.127 (0.124)	-0.125 (0.125)
**Depressive symptoms**	0.009 (0.003) ***	-0.011 (0.006) *	-0.011 (0.006) *		-0.010 (0.027)	0.012 (0.036)
N	4396					

Notes: ADL= activities of daily living. FE= Fixed effects. Cells represents coefficient (standard error). *** p<0.01, ** p<0.05, * p<0.1.

Supplementary Table 5. The relationship between informal care intensity and health among older people in China

	Number of ADL limitations			Depressive symptoms		
**Variables**	**FE model**	**Lagged FE model**	**Lagged FE model with interaction**	**FE model**	**Lagged FE model**	**Lagged FE model with interaction**
**Hours of informal care (ln)**	0.295 (0.054) ***	-0.293 (0.195)	-0.291 (0.210)	0.161 (0.128)	-0.807 (0.453) *	-0.563 (0.377)
**Hours of informal care (ln)* Income (ln)**						
Hours of informal care (ln) *Lowest quintile			Ref			Ref
Hours of informal care (ln)*Second quintile			0.238 (0.883)			1.825 (2.349)
Hours of informal care (ln)*Middle quintile			0.476 (1.007)			3.492 (4.879)

Hours of informal care (ln)*Fourth quintile			0.422 (0.930)			4.311 (4.826)
Hours of informal care (ln)*Top quintile			-0.043 (0.851)			3.257 (3.896)
**Income**						
Lowest quintile	Ref	Ref	Ref	Ref	Ref	Ref
Second quintile	-0.113 (0.248)	0.278 (0.739)	1.398 (1.455)	-0.124 (0.560)	0.314 (1.570)	0.869 (1.370)
Middle quintile	-0.016 (0.237)	0.205 (0.761)	0.797 (0.989)	-0.315 (0.536)	0.314 (1.570)	1.664 (1.462)
Fourth quintile	-0.311 (0.261)	0.050 (0.909)	1.037 (1.134)	0.124 (0.591)	1.367 (1.750)	1.011 (1.392)
Top quintile	0.028 (0.286)	-1.436 (0.900)	0.709 (0.982)	0.199 (0.646)	0.903 (1.658)	-2.129 (1.494)
**Age**						
65-80	Ref	Ref	Ref	Ref	Ref	Ref

80+	0.240 (0.386)	-0.491 (1.434)	-0.388 (1.549)	-0.708 (0.872)	3.752 (2.929)	3.762 (1.867) **
**Gender**						
Female	Omitted	Ref	Ref	Omitted	Ref	Ref
Male	Omitted	-0.150 (0.375)	-0.090 (0.488)	Omitted	-0.931 (1.100)	-2.595 (1.499) *
**Education attainment**						
Illiteracy	Omitted	Ref	Ref	Omitted	Ref	Ref
Elementary school	Omitted	0.176 (0.674)	-0.028 (0.576)	Omitted	1.488 (1.217)	3.214 (1.666) *
Middle school and above	Omitted	1.009 (0.835)	0.218 (0.399)	Omitted	0.000 (1.923)	0.704 (2.348)
**Marital status**						
Others	Ref	Ref	Ref	Ref	Ref	Ref
Widowed	-0.220 (0.706)	1.914 (1.734)	3.431 (2.072) *	0.858 (1.593)	1.834 (1.745)	0.081 (1.121)
Married	0.015 (0.677)	2.650 (1.905)	1.85 (2.185)	-0.639 (1.529)	2.692 (1.786)	-1.344 (2.346)
**Residence**						
City	Ref	Ref	Ref	Ref	Ref	Ref
Town	-0.855 (0.395) **	-0.812 (0.780)	-0.939 (1.029)	0.313 (0.899)	1.332 (0.908)	0.965 (0.643)

Rural	-0.884 (0.390) **	-0.582 (0.747)	-0.617 (0.817)	0.356 (0.888)	-0.459 (1.433)	-0.042 (0.995)
**Living with family members**						
No	Ref	Ref	Ref	Ref	Ref	Ref
Yes	0.374 (0.313)	-0.718 (1.062)	-0.299 (0.925)	-0.016 (0.708)		1.964 (1.209)
**Smoking**						
No	Ref	Ref	Ref	Ref	Ref	Ref
Yes	-0.311 (0.376)	-0.432 (1.170)	-0.191 (1.177)	0.555 (0.850)	-1.987 (2.195)	-0.661 (1.586)
**Drinking**						
No	Ref	Ref	Ref	Ref	Ref	Ref
Yes	0.124 (0.358)	0.288 (0.742)	0.620 (0.838)	-1.166 (0.806)	-0.303 (1.142)	-0.536 (1.491)
**Self-rated health**						
Bad	Ref	Ref	Ref	Ref	Ref	Ref
Fair	-0.231 (0.205)	0.288 (0.742)	0.513 （0.664）	-0.529 (0.464)	1.208 (1.100)	2.475 (0.902) ***
Good	-0.414 (0.234) *	0.683 (0.499)	1.219 （0.496） **	-2.285 (0.514) ***	2.365 (1.011) ***	3.326 (1.864) ***

**Number of chronic diseases**	0.045 (0.058)	-0.076 (0.117)	0.135 (0.708)	0.273 (0.130) **	0.072 (0.124)	0.087 (0.253)
**Cognitive function scores**	-0.026 (0.013) **	0.024 (0.017)	0.065 (0.138)	-0.047 (0.029) *	-0.033 (0.055)	-0.008 (0.065)
**Number of ADL limitations**		0.670 (0.305)	0.845 (0.131) ***	0.236 (0.134) *	-0.824 (0.380) **	-1.080 (0.513) **
**Depressive symptoms**	0.046 (0.026) *	0.101 (0.038) ***	0.119 (0.172)		0.321 (0.247)	0.079 (0.172)
N				1,687		

Notes: ADL= activities of daily living. FE= Fixed effects. Cells represents coefficient (standard error). *** p<0.01, ** p<0.05, * p<0.1.

Supplementary Table 6. The relationship between informal care intensity and health among older people in China

	Number of ADL limitations			Depressive symptoms		
**Variables**	**FE model**	**Lagged FE model**	**Lagged FE model with interaction**	**FE model**	**Lagged FE model**	**Lagged FE model with interaction**
**Receiving intensive informal care**	0.733 (0.158) ***	-0.287 (0.315)	2.475 (2.442)	0.424 (0.365)	-0.387 (0.786)	-6.917 (4.771)
**Receiving intensive informal care* Income (ln)**			-0.340 (0.300)			0.795 (0.572)
**Income (ln)**	-0.066 (0.062)	0.072 (0.113)	0.295 (0.231)	0.083 (0.138)	-0.639 (0.289) **	-1.161 (0.465) **
**Age**						
65-80	Ref	Ref	Ref	Ref	Ref	Ref
80+	0.392 (0.388)	-0.245 (0.760)	-0.099 (0.821)	-0.633 (0.866)	3.734 (1.980) *	3.079 (1.926)
**Gender**						
Female	Omitted	Ref	Ref	Omitted	Ref	Ref
Male	Omitted	-0.138 (0.754)	0.063 (0.668)	Omitted	-2.236 (1.333) *	-2.125 (1.326)
**Education attainment**						
Illiteracy	Omitted	Ref	Ref	Omitted	Ref	Ref
Elementary school	Omitted	0.211 (0.747)	0.075 (0.684)	Omitted	2.781 (1.492) *	2.962 (1.483) **

Middle school and above	Omitted	0.598 (1.064)	0.309 (0.951)	Omitted	-0.311 (2.204)	0.216 (2.192)
**Marital status**						
Others	Ref	Ref	Ref	Ref	Ref	Ref
Widowed	-0.146 (0.710)	2.555 (2.029)	4.098 (4.375)	0.852 (1.584)	-0.553 (1.657)	0.517 (1.347)
Married	0.024 (0.682)	2.515 (2.195)	2.005 (3.432)	-0.755 (1.520)	-0.703 (1.489)	0.783 (1.658)
**Residence**						
City	Ref	Ref	Ref	Ref	Ref	Ref
Town	-0.928 (0.395) **	-0.362 (0.993)	-1.041 (1.641)	0.315 (0.890)	0.982 (0.623)	1.241 (1.740)
Rural	-0.891 (0.391) **	-0.254 (1.146)	-0.821 (1.328)	0.426 (0.880)	-1.207 (2.328)	0.082 (1.148)
**Living with family members**						
No	Ref	Ref	Ref	Ref	Ref	Ref
Yes	0.393 (0.312)	0.214 (0.533)	0.233 (0.567)	0.048 (0.699)	2.326 (1.294) *	1.965 (1.260)
**Smoking**						
No	Ref	Ref	Ref	Ref	Ref	Ref

Yes	-0.251 (0.382)	-0.779 (1.535)	-0.423 (1.078)	0.565 (0.851)	-1.768 (2.257)	0.087 (2.034)
**Drinking**						
No	Ref	Ref	Ref	Ref	Ref	Ref
Yes	0.084 (0.360)	0.309 (0.850)	0.335 (0.725)	-1.258 (0.800)	-0.442 (1.538)	-0.511 (1.580)
**Self-rated health**						
Bad	Ref	Ref	Ref	Ref	Ref	Ref
Fair	-0.336 (0.206)	0.466 (0.518)	0.121 (0.448)	-0.636 (0.460)	1.299 (1.323)	2.845 (1.360) **
Good	-0.501 (0.234) **	1.226 (0.520) **	0.893 (0.474) *	-2.419 (0.505) ***	2.718 (1.816)	3.955 (1.306) ***
**Number of chronic diseases**	0.070 (0.058)	-0.099 (0.121)	-0.061 (0.135)	0.276 (0.129) **	0.231 (0.308)	0.152 (0.301)
**Cognitive function scores**	-0.030 (0.013) **	0.021 (0.029)	0.015 (0.031)	-0.044 (0.028)	-0.060 (0.073)	-0.048 (0.071)
**Number of ADL limitations**		0.412 (0.428)	0.701 （0.552）	0.251 (0.131) *	-0.886 (0.511) *	-1.116 (0.524)
**Depressive symptoms**	0.050 (0.026_ *	0.049 (0.051)	0.030 (0.058)		0.114 (0.169)	0.113 (0.163)

N	1,687

Notes: ADL= activities of daily living. FE= Fixed effects. Cells represents coefficient (standard error). *** p<0.01, ** p<0.05, * p<0.1.
